# European Survey on Clinical Practice of Detecting and Treating T-Cell Mediated Kidney Transplant Rejection

**DOI:** 10.3389/ti.2024.12283

**Published:** 2024-04-18

**Authors:** Priyanka Koshy, Lucrezia Furian, Peter Nickerson, Gianluigi Zaza, Maria Haller, Aiko P. J. de Vries, Maarten Naesens

**Affiliations:** ^1^ Department of Microbiology, Immunology and Transplantation, KU Leuven, Leuven, Belgium; ^2^ Department of Pathology, University Hospitals Leuven, Leuven, Belgium; ^3^ Kidney and Pancreas Transplantation Unit, Department of Surgical Gastroenterological and Oncological Sciences, University Hospital of Padua, Padua, Italy; ^4^ Department of Internal Medicine, University of Manitoba, Winnipeg, MB, Canada; ^5^ Renal, Dialysis and Transplant Unit, Department of Medical and Surgical Sciences, University of Foggia, Foggia, Italy; ^6^ Section for Clinical Biometrics, Center for Medical Statistics, Informatics and Intelligent Systems (CeMSIIS), Medical University of Vienna, Vienna, Austria; ^7^ Nephrology, Ordensklinikum Linz, Elisabethinen, Linz, Austria; ^8^ Department of Medicine, Division of Nephrology, Leiden Transplant Center, Leiden University Medical Center, Leiden, Netherlands; ^9^ Department of Nephrology and Kidney Transplantation, University Hospitals Leuven, Leuven, Belgium

**Keywords:** survey, clinical practice, therapy, diagnostics, TCMR

## Abstract

The KDIGO guideline for acute rejection treatment recommends use of corticosteroids and suggests using lymphocyte-depleting agents as second line treatment. Aim of the study was to determine the current practices of detection and treatment of TCMR of kidney allografts amongst European kidney transplant centres. An invitation was sent through ESOT/EKITA newsletters and through social media to transplant professionals in Europe for taking part in the survey. A total of 129 transplant professionals responded to the survey. There was equal representation of small and large sized transplant centres. The majority of centres treat borderline changes (BL) and TCMR (Grade IA-B, IIA-B) in indication biopsies and protocol biopsies with corticosteroids as first line treatment. Thymoglobulin is used mainly as second line treatment for TCMR Grade IA-B (80%) and TCMR IIA-B (85%). Treatment success is most often evaluated within one month of therapy. There were no differences observed between the large and small centres for the management of TCMR. This survey highlights the common practices and diversity in clinics for the management of TCMR in Europe. Testing new therapies for TCMR should be in comparison to the current standard of care in Europe. Better consensus on treatment success is crucial for robust study designs.

## Graphical Abstract

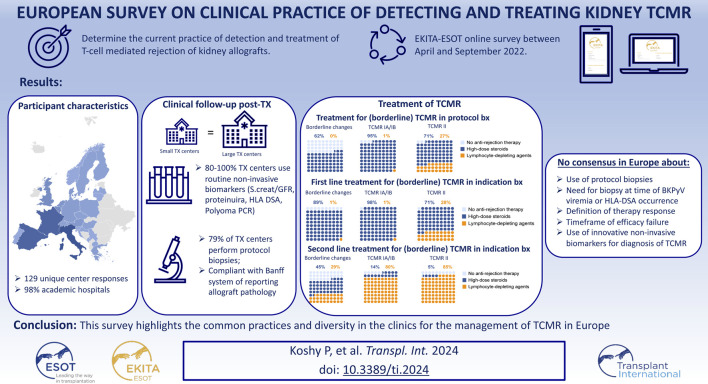

## Introduction

One of the major causes of graft failure is alloimmune rejection, either T cell-mediated, antibody-mediated, or mixed [1, 2]. The histopathological diagnosis of allograft rejection is established by following the Banff working scheme [3–5], which has undergone periodic revisions, based on immunological and clinical insights, clinical and epidemiological studies, and emerging trends of molecular diagnostics.

Despite the progress in precision diagnostics of allograft rejection, very little progress has been made in therapeutics. While the past two decades have seen several attempts to establish the treatment for antibody-mediated rejection (AMR) [6], lesser studies have evaluated treatment options for T cell-mediated rejection (TCMR) [7]. Thymoglobulin, the last drug approved for treatment of TCMR, was approved in 1998. A systematic review indicated that antibody therapy was probably better than steroids in reversing acute cellular rejection and in preventing subsequent rejection, and also in preventing graft loss. T cell depleting antibodies are efficacious but associated with a much greater risk for adverse effects [8, 9]. However, no information is available on rejection grades or clinical context; most studies were performed only with rejection in indication biopsies. Few clinical trials on treatment for subclinical TCMR with steroids showed mixed results [10–12]. Since the T-cell depleting agents were approved, no new drugs were studied for this indication, despite the high unmet need for effective treatment of TCMR, with less therapeutic side effects.

The 2009 KDIGO guideline for treatment of acute TCMR recommends the use of corticosteroids as the initial treatment and suggests using lymphocyte-depleting antibodies (ATG or thymoglobulin; OKT3 is no longer available) if the patient is non-responsive to corticosteroids or if there is recurrence of acute cellular rejection. It was also suggested that subclinical and borderline TCMR should be treated and background immunosuppression optimized [13]. More recent guidelines echo these recommendations, without further evidence supporting them, also acknowledging that the use of protocol biopsies to detect and treat subclinical rejection is not built on strong evidence [14].

Because of both the lack of strong evidence for treatment choices in subtypes or different grades of (borderline) TCMR and the absence of international consensus guidelines on this topic, transplantation centre practices differ substantially. Not only do transplantation centre practices differ in the performance of protocol biopsies [6], but also in the treatment approaches for patients with rejection as reported in study reports on this topic [7]. Surveys in the United States and Canada confirmed this heterogeneity and indicate also differences between countries [15, 16]. Recent reports, on the background of tacrolimus-mycophenolate based therapy, document a high rate of persistent rejection following anti-rejection therapy for both clinical and subclinical rejection, which is associated with poor long-term outcomes (i.e., *de novo* anti-HLA donor-specific antibodies, AMR graft loss) [17, 18].

The last consensus forum defining efficacy endpoints for the assessment of anti-rejection therapy was in 1995 and relied primarily on renal functional criteria [19]. The definitions of rejection, insights in pathophysiology and outcome, and treatment options have changed significantly over the past 25 years. Therefore, a new consensus on more recent data is needed. However, European data on the current clinical practice of detection, treatment, and follow-up after rejection are lacking.

As the clinical practice in Europe is likely different from that in Canada and the United States, enriching the debate and adapting consensus to the current European reality is necessary. Charting the standard of care in clinical practice is essential in designing innovator drug trials, which need a well-defined comparator group. Insight in current routine practice of TCMR diagnosis and treatment could pave the way to new trials heavily needed in the field.

Here, we report on a survey conducted to determine the current practices of detection and treatment of TCMR of kidney allografts amongst European kidney transplant centres, and compare these practices with previous reports from the United States and Canada [15, 16].

## Methods

A survey was drafted by all co-authors and transferred to a SurveyMonkey (Momentive Global Inc., San Mateo, California, United States) web-based platform, which was tested by all co-authors. An invitation to participate in the survey was sent through the European Society for Organ Transplantation (ESOT) and European Kidney Transplant Association (EKITA) newsletters and through a social media campaign to transplant professionals in Europe for taking part in the survey. Several reminders were sent. Also, an individual email campaign was launched to reach as many centres as possible in Europe. The survey was conducted in 2022.

The survey questionnaire was divided into two parts.

Part 1 consisted of four categories:

- Category 1—Survey participant characteristics—questions regarding specialization, population treated, years in practice, type of transplantation centre, size of centre, induction therapy at time of transplantation, time period of steroid withdrawal, percentage of living donors and percentage of repeat transplants.
- Category 2—Clinical follow-up post-transplant—questions regarding clinical follow-up by whom, where, performance of protocol biopsies, indications for for-cause biopsies and about non-invasive testing to guide kidney transplant biopsies.
- Category 3—Diagnosis of rejection—questions regarding reporting of allograft biopsies, use of Banff lesion scores, diagnosis of rejection without performing kidney biopsy, use of molecular microscope for diagnosis of rejection in routine clinic, rate of clinical TCMR, definition of borderline rejection, authority approval of thymoglobulin and alemtuzumab.
- Category 4—Definition of successful rejection treatment of TCMR.

Part 2 consisted of questions on treatment of subclinical and clinical TCMR.

Descriptive statistical analyses were performed in Prism 9 for macOS (GraphPad Prism version 9.5.0, GraphPad Software, San Diego, California United States^1^).

## Results

### Survey Participant Characteristics

**FIGURE 1:**
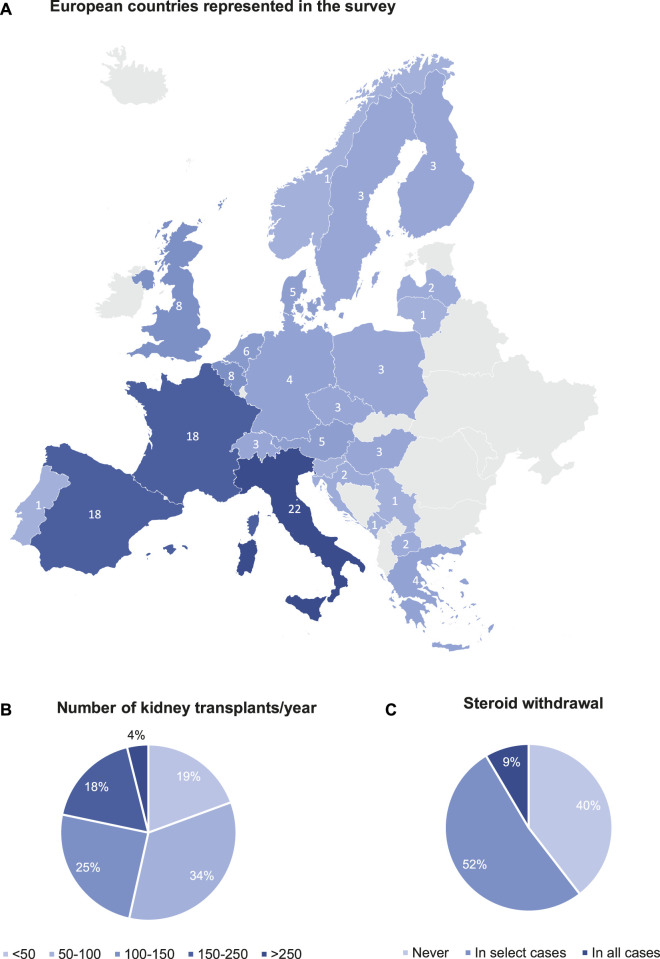
Survey participant characteristics. **(A)** European countries represented in the survey. **(B)** Number of kidney transplants/year. **(C)** Steroid withdrawal.

Survey participant characteristics are detailed in Table 1. A total of 129 European transplant professionals representing 25 European countries responded to the survey (Figure 1A). Most of the participants were transplant nephrologists (78.1%) treating the adult population with more than 11 years of experience. 94 (72.9%) participants volunteered to mention their affiliation, and they represent 92 major university hospitals in Europe. 85.9% of centres perform uniquely adult kidney transplants, 10.2% both adults and paediatric transplants, and 3.9% in children/adolescents only. 69 (53.5%) transplant centres perform <100 kidney transplantations per year, while 60 (46.5%) transplant centres perform >100 kidney transplantations per year on average (Figure 1B). Living donation rates vary greatly between centres and countries. The majority (69.5%) perform 11%–25% repeat transplantations. It has not been surveyed whether induction therapy is used in all or selected patients. There is a heterogeneity in the drugs used for induction therapy at the time of transplantation (Table 2): 72% of the respondents use either basiliximab or thymoglobulin; 8% of the respondents include alemtuzumab in their armamentarium for induction. Many respondents (51.9%) stop administering steroids within the first months after transplantation in selected cases (not further specified), while other respondents (39.5%) do not have steroid withdrawal protocols. Only few respondents (8.5%) systematically discontinue steroids in all cases within the first months after transplantation (Figure 1C).

**TABLE 1:** Participant characteristics.

Question	Multiple choices	Number of centres (*N*)	Percentages (%)
**Specialization**	Nephrologist	100	78.1%
(*n* = 128)	Transplant surgeon	21	16.4%
[1 participant did not respond to this question]	Pathologist	3	2.3%
	Others (transplant coordinator, immunologist, intensivist)	4	3.1%
**Population treated**	Adult	110	85.9%
(*n* = 128)	Paediatric	5	3.9%
[1 participant did not respond to this question]	Adult and paediatric	13	10.2%
**Years in practice**	Still in training	6	4.7%
(*n* = 127)	<5 years	16	12.6%
[2 participants did not respond to this question]	5–10 years	19	15.0%
	11–20 years	39	30.7%
	>20 years	47	37.0%
**Type of centre**	Academic	125	97.7%
(*n* = 128)	Private	1	0.8%
[1 participant did not respond to this question]	Others (public hospital, non-benefit pvt hospital)	2	1.6%
**Size of centre**	<50 kidney transplantations/year	25	19.4%
(*n* = 129)	50–100 kidney transplantations/year	44	34.1%
	100–150 kidney transplantations/year	32	24.8%
	150–250 kidney transplantations/year	23	17.8%
	>250 kidney transplantations/year	5	3.9%
**Living donor %**	<10%	30	23.3%
(*n* = 129)	10–<25%	58	45.0%
	>25%	41	31.8%
**Repeat transplants %**	<10%	24	18.8%
(*n* = 128)	11%–25%	89	69.5%
[1 participant did not respond to this question]	25%–50%	15	11.7%
	>50%	0	0

**TABLE 2:** Standard of care therapy for kidney transplantation—induction and treatment for TCMR other than steroids.

Question	Multiple choices	Number of centres (*N*)	Percentages (%)
**Type of induction therapy used at the time of transplantation**	Basiliximab	20	16.0
(*n* = 125)	Thymoglobulin/ATG	5	4.0
[4 participants did not respond to this question]	Alemtuzumab	1	0.8
	Basiliximab or Thymoglobulin/ATG	90	72.0
	Basiliximab or Alemtuzumab	7	5.6
	Basiliximab or Thymoglobulin/ATG or Alemtuzumab	1	0.8
	Thymoglobulin/ATG or Alemtuzumab	1	0.8
**Steroid withdrawal within the first months after transplantation**	Yes, in all cases	11	8.5
(*n* = 129)	Yes, in select cases	67	51.9
	No	51	39.5
**Authority approval of thymoglobulin/ATG in kidney transplantation—all that apply**	For treatment of rejection, without specification, to be decided by the treating physician	89	74.2
(*n* = 120)	Only for treatment of steroid-resistant rejection	32	26.7
[9 participants did not respond to this question]	Only in case of rejection at time of graft dysfunction (indication biopsies)	6	5.0
	Only as induction therapy	77	64.2
	There is no reimbursement	1	0.8
	Other (desensitization, as primary treatment for TCMR - Grade 2a upward, steroid resistant rejection, v > 0)	3	2.5
**Availability of alemtuzumab for treatment of rejection**	For treatment of rejection, without specification, to be decided by the treating physician	19	17.0
(*n* = 112)	For treatment of steroid-resistant rejection	7	6.2
[17 participants did not respond to this question]	Not available for treatment of rejection	86	76.8

### Clinical Follow-Up Post-Transplant

**FIGURE 2:**
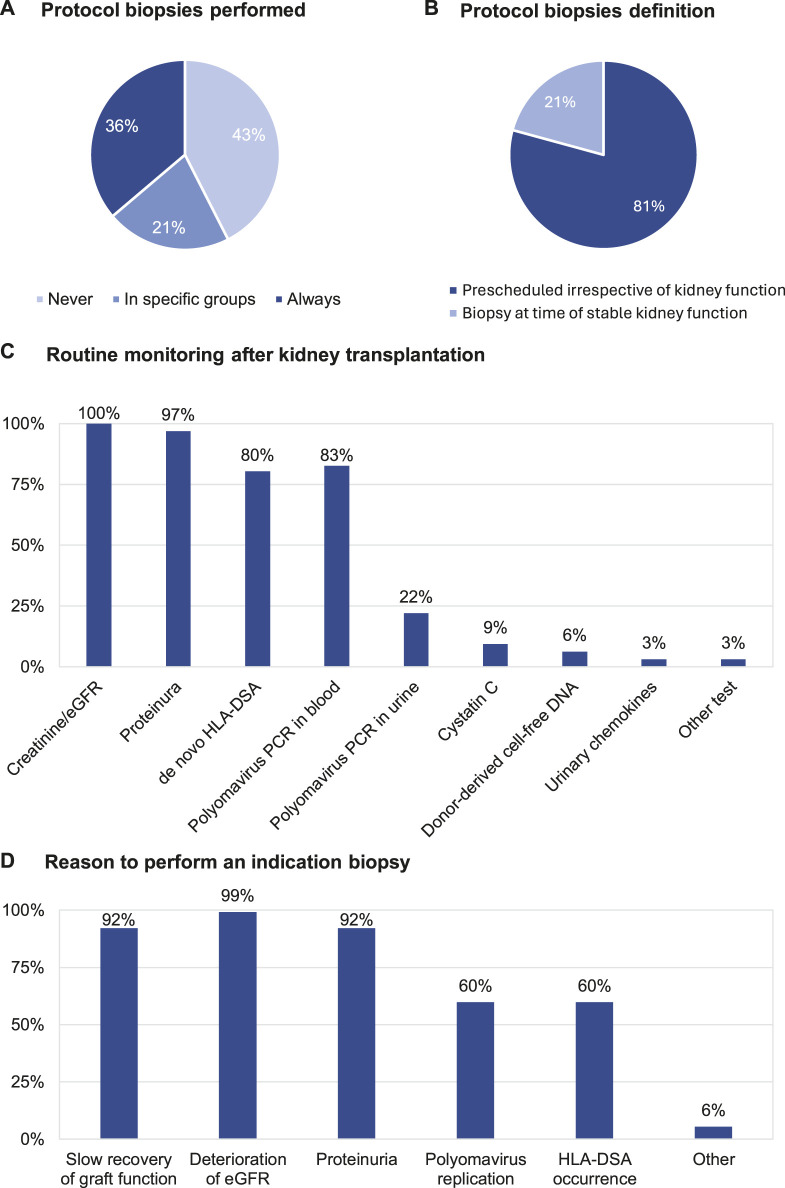
Clinical follow-up post-transplant: **(A)** Protocol biopsies performed. **(B)** Protocol biopsies definition. **(C)** Routine monitoring after kidney transplantation. **(D)** Reason to perform an indication biopsy.

Table 3 summarizes the standard practices for post-transplant follow-up by the respondents included in the survey. The clinical follow-up post-transplant is conducted mainly by the transplant nephrologists (92.1%) in the transplant centre (62.2%) but hybrid follow-up in collaboration with the referring centre is also common (31.5%). Protocol biopsies are performed in the centres of 57.5% of respondents (Figure 2A), but only 36.2% of respondents always perform a protocol biopsy. 21.3% of respondents perform protocol biopsies in specific subgroups of patients, for example, in highly sensitized/immunized patients; in patients with positive donor-specific anti-HLA antibodies (HLA-DSA); in patients participating in clinical trials; and depending on the primary native kidney disease. Protocol biopsies are mainly conducted at 3 months and 1 year post-transplant; very few respondents perform protocol biopsies later after transplantation. There is no difference in performing protocol biopsies between the respondents performing <100 renal transplantations/year and the respondents performing >100 renal transplantations/year (Supplementary Table S1). Most of the respondents performing protocol biopsies (81.3%), defined protocol biopsies as “prescheduled, irrespective of kidney function (Figure 2B).” 55.9% of respondents perform kidney biopsies after hospitalization of patients and 44.1% respondents perform kidney biopsies as outpatient procedure. The routine non-invasive testing to guide kidney transplant biopsies are serum creatinine/eGFR (100%), proteinuria (96.9%), polyomavirus PCR in blood (82.7%), monitoring for *de novo* HLA-DSA (80.3%) and polyomavirus PCR in urine (22%). Only a few respondents (<10%) also monitor cystatin C (9.4%), urinary chemokines (3.1%), and donor derived cell-free DNA (6.3%) (Figure 2C). The common indications for “for-cause” biopsies are slow recovery of graft function (92.1%), deterioration of eGFR (99.2%), and proteinuria (92.1%). There is less concordance about performing an indication biopsy at the time of polyomavirus replication (59.8%) or with HLA-DSA occurrence (59.8%) (Figure 2D).

**TABLE 3:** Clinical follow-up post-transplant.

Question	Multiple choices	Number of centres (*N*)	Percentages (%)
**By whom**	Nephrologist	117	92.1
(*n* = 127)	Transplant surgeon	4	3.1
[2 participants did not respond to this question]	Others (both)	6	4.7
**Where**	Transplant centre	79	62.2
(*n* = 127)	Referring centre	5	3.9
[2 participants did not respond to this question]	Mixed/Hybrid	40	31.5
	Others	3	2.4
**Protocol biopsies performed**	Never	54	42.5
(*n* = 127)	Always	46	36.2
[2 participants did not respond to this question]	In specific groups	27	21.3
**Definition of protocol biopsies**	Prescheduled, irrespective of kidney function	87	81.3
(*n* = 107) [22 participants did not respond to this question]	Defined based on stable kidney function	20	18.7
**Timing of protocol biopsies—all that apply**	1 week	1	1.4
(*n* = 73)	2 weeks	1	1.4
	1 month	5	6.8
	3 months	48	65.8
	6 months	8	11.0
	1 year	45	61.6
	2 years	9	12.3
	5 years	2	2.7
	10 years	1	1.4
	Others (3 years)	3	4.1
**Standard biopsy procedure**	Hospitalization	71	55.9
(*n* = 127) [2 participants did not respond to this question]	Outpatient based	56	44.1
**Indications for “for-cause” biopsies—all that apply**	Slow recovery of graft function	117	92.1
(*n* = 127)	Deterioration of eGFR	126	99.2
[2 participants did not respond to this question]	Proteinuria	117	92.1
	Polyomavirus replication	76	59.8
	HLA-DSA occurrence	76	59.8
	Others	7	5.5
**Routine non-invasive testing to guide kidney transplant biopsies—all that apply**	Serum creatinine/eGFR	127	100
(*n* = 127)	Proteinuria	123	96.9
[2 participants did not respond to this question]	Cystatin C	12	9.4
	Polyomavirus PCR in urine	28	22.0
	Polyomavirus PCR in blood	105	82.7
	Urinary chemokines	4	3.1
	Donor-derived cell-free DNA testing	8	6.3
	Monitoring for *de novo* HLA-DSA occurrence	102	80.3
	Other tests (CMV, non-HLA antibody testing, MAG3 at DGF, DSA for high risk cases only)	4	3.1

### Diagnosis of TCMR

In Europe, the kidney transplant biopsies are mostly evaluated by renal pathologists (91.7%), who are considered to follow the most recent Banff 2019 classification (Table 4). Most of the pathology reports (90.8%) include the individual Banff lesion scores routinely. Many respondents (64.5%) never diagnose rejection without performing a kidney biopsy, but this is not universal and 24% of respondents diagnose rejection based on non-invasive markers not always confirmed by tissue biopsy. Most respondents (93.4%) do not use biopsy-based molecular diagnostics for the diagnosis of rejection in routine clinical practice.

**TABLE 4:** Diagnosis of rejection.

Question	Multiple choices	Number of centres (*N*)	Percentages (%)
**Biopsy results evaluated by**	Nephropathologist	111	91.7
(*n* = 121)	General pathologist	7	5.8
[8 participants did not respond to this question]	Nephrologist	3	2.5
**Pathology report—definition of TCMR**	According to the most recent Banff 2019 classification	117	96.7
(*n* = 121)	According to older versions of Banff classification	2	1.7
[8 participants did not respond to this question]	Not according to Banff classification	2	1.7
**Pathology report—individual lesion scores**	Individual Banff lesion scores are routinely reported	109	90.8
(*n* = 120) [9 participants did not respond to this question]	Individual Banff lesion scores are not routinely reported	11	9.2
**Diagnosis of rejection without performing a kidney transplant biopsy**	Never	78	64.5
(*n* = 121)	Based on non-invasive markers but not always confirmed by biopsy	29	24.0
[8 participants did not respond to this question]	We do not do biopsies to confirm rejection	1	0.8
	Others (in patients with high risk/contraindication)	13	10.7
**Molecular microscope for diagnosis of rejection in routine clinic**	Never	113	93.4
(*n* = 121)	Always	1	0.8
[8 participants did not respond to this question]	In specific cases (mainly for clinical trials/research)	7	5.8
**Rate of clinical TCMR (in indication biopsies)**	<5%	15	12.8
(*n* = 117)	5-<11%	39	33.3
[12 participants did not respond to this question]	11–<16%	30	25.6
	16-<26%	23	19.7
	>26%	10	8.5
**Definition of borderline changes**	t ≥ 1, i ≥ 1 threshold	73	60.3
(*n* = 121)	t 1/2/3 with i0 considered as borderline changes	21	17.4
[8 participants did not respond to this question]	Other (t1 or t0 with i1 or i0)	1	0.8
	Unknown	26	21.5

The rate of clinical TCMR in indication biopsies reported by the respondents is highly variable, and significantly relates to the rate of repeat transplantations (Supplementary Table S2). Most respondents (60.3%) report using the Banff 2019 (t ≥ 1, i ≥ 1) threshold for the definition of borderline changes in their centre, but 26 respondents (21.5%) do not know the threshold used at their centre for defining borderline changes.

### Definition of Successful Rejection Treatment of TCMR

**FIGURE 3:**
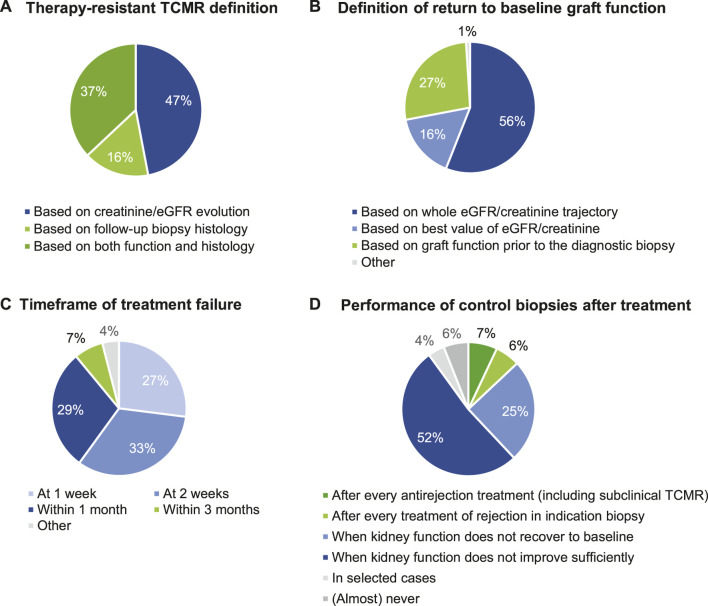
Definition of successful rejection treatment of TCMR. **(A)** Therapy-resistant TCMR definition. **(B)** Definition of return to baseline graft function. **(C)** Timeframe of treatment failure. **(D)** Performance of control biopsies after treatment.

We next evaluated the definitions of “successful rejection treatment.” The definition of therapy resistant TCMR is highly heterogeneous (Table 5). The question asked to the participants (“all that apply”) lead to redundancy in the responses, as several respondents ticked multiple choices—“When creatinine/eGFR does not completely return to baseline”; “When creatinine/eGFR recovers not at all or at best partly” and “When creatinine/eGFR does not improve anything.” This indicates that the definitions of complete return to baseline, partial recovery or “any improvement” are unclear to the respondents. Therefore, we reformatted the responses to evaluate whether creatinine/eGFR vs. histological evaluations was considered for the definition of therapy resistant TCMR. This indicates high heterogeneity in this definition, with 47% of respondents using creatinine/eGFR evolution, 16% pure biopsy histology, and 37% integration of information from biopsies and from creatinine/eGFR for the definition of therapy resistance; 53% of respondents integrate the use of a repeat biopsy in the definition of therapy resistance (Figure 3A).

**TABLE 5:** Definition of successful rejection treatment of TCMR.

Question	Multiple choices	Number of centres (*N*)	Percentages (%)
**Definition of “therapy resistant TCMR”—all that apply**	When creatinine/eGFR does not completely return to baseline	35	29.9
(*n* = 117)	When creatinine/eGFR recovers not at all or at best partly	59	50.4
[12 participants did not respond to this question]	When creatinine/eGFR does not improve anything	44	37.6
	Based on follow-up biopsy histology	62	53.0
	Others	4	3.4
**Definition of “therapy resistant TCMR”**	Based on graft functional evolution	55	47.0
(*n* = 117)	Based on follow-up biopsy histology	19	16.2
[12 participants did not respond to this question]	Based on combination of functional evolution and follow-up biopsy histology	43	36.8
**Definition of “steroid-resistant TCMR”**	When creatinine/eGFR does not completely return to baseline after high-dose steroid treatment	29	25.0
(*n* = 116)	When creatinine/eGFR recovers not at all or at best partly after highdose steroid treatment	42	36.2
[13 participants did not respond to this question]	When creatinine/eGFR does not improve anything	10	8.6
	Based on follow-up biopsy histology	28	24.1
	When second-line therapy is initiated, irrespective of kidney function or histology	4	3.4
	Other	3	2.6
**Definition of “return to baseline kidney transplant function”**	Based on whole eGFR/creatinine trajectory	66	56.4
(*n* = 117)	Based on best value of eGFR/creatinine	19	16.2
[12 participants did not respond to this question]	Based on graft function prior to the diagnostic biopsy	31	26.5
	Other	1	0.8
**Timeframe of efficacy failure of antirejection treatment**	At 1 week	30	26.5
(*n* = 113)	At 14 days	37	32.7
[16 participants did not respond to this question]	Within 1 month	33	29.2
	Within 3 months	8	7.1
	Within 6 months	0	0
	Others	5	4.4
**Performance of a control/follow-up biopsy after rejection treatment to see disease resolution**	After every antirejection treatment, also when diagnosed in protocol biopsies	8	6.8
(*n* = 117)	After every treatment for clinical TCMR, also when kidney function improved	7	6.0
[12 participants did not respond to this question]	When kidney function did not completely recover to baseline	29	24.8
	When renal function did not improve sufficiently upon treatment	61	52.1
	In selected cases	5	4.3
	(Almost) never	7	6.0
**If control biopsies are performed, when are they planned**	After 14 days	29	29.3
(*n* = 99)	After 1 month	23	23.2
[30 participants did not respond to this question]	After 3 months	18	18.2
	After 6 months	3	3.0
	Others	26	26.3

The majority of respondents define “steroid resistant TCMR” based on graft functional characteristics (36.2% when creatinine/eGFR recovers not at all or at best partly; 25.0% when creatinine/eGFR does not completely return to baseline; 8.6% when creatinine/eGFR does not improve anything), but 24.1% indicate defining this based on follow-up biopsy histology; combinations between graft functional and histological definition were not allowed for this question (Table 5).

The majority of respondents define “return to baseline kidney transplant function” by assessing the whole eGFR/creatinine trajectory (56.4%), while others base this evaluation on graft function prior to the diagnostic biopsy (26.5%) and based on the best value of eGFR/creatinine (16.2%), again indicating lack of consensus in these responses (Table 5; Figure 3B).

Next, we surveyed the timeframe of efficacy failure of antirejection treatment. Most respondents (88.5%) consider therapy failure “within 1 month” as the period of efficacy failure of antirejection treatment. Only 7.1% of respondents consider therapy failure at 3 months or later (Table 5; Figure 3C). Many respondents (76.9%) perform a control/follow-up biopsy after rejection treatment for assessment of disease resolution only when the renal function does not improve sufficiently upon treatment; systematic control biopsies are performed in only 12.8% (Table 5; Figure 3D). If control biopsies are performed after rejection treatment, their timing is very variable between respondents; either after 14 days (29.3%), after 1 month (23.2%), or after 3 months (18.2%); others responded that this timing depends on kidney functional evolution. Altogether, this indicates that there is no consensus on the best timing for performing a control biopsy (Table 5).

### Treatment of TCMR

The responses to the questions about first-line and second-line treatment for TCMR were highly variable between respondents. The granular responses are summarized by counting the strongest therapy indicated by the respondent for each rejection type (Thymoglobulin/ATG/alemtuzumab > IV steroids with PO taper > high-dose IV steroids > PO steroid taper > increase baseline immunosuppression > no change). Several centres report, e.g., combinations of ATG with IV steroids and increase baseline immunosuppression. Doses of IV corticosteroids range between 250, 500 and 1,000 mg for 3 days. PO steroid taper was not further specified.

Most centres (74.2%) report having authority approval for using thymoglobulin/ATG at the physician’s discretion, while others (23.7%) can use thymoglobulin/ATG only for treatment of steroid-resistant rejection. Alemtuzumab is not widely available in Europe; only 23.2% of centres report having access for anti-rejection treatment (Table 2).

#### Subclinical (Borderline) TCMR in Protocol Biopsies

**FIGURE 4:**
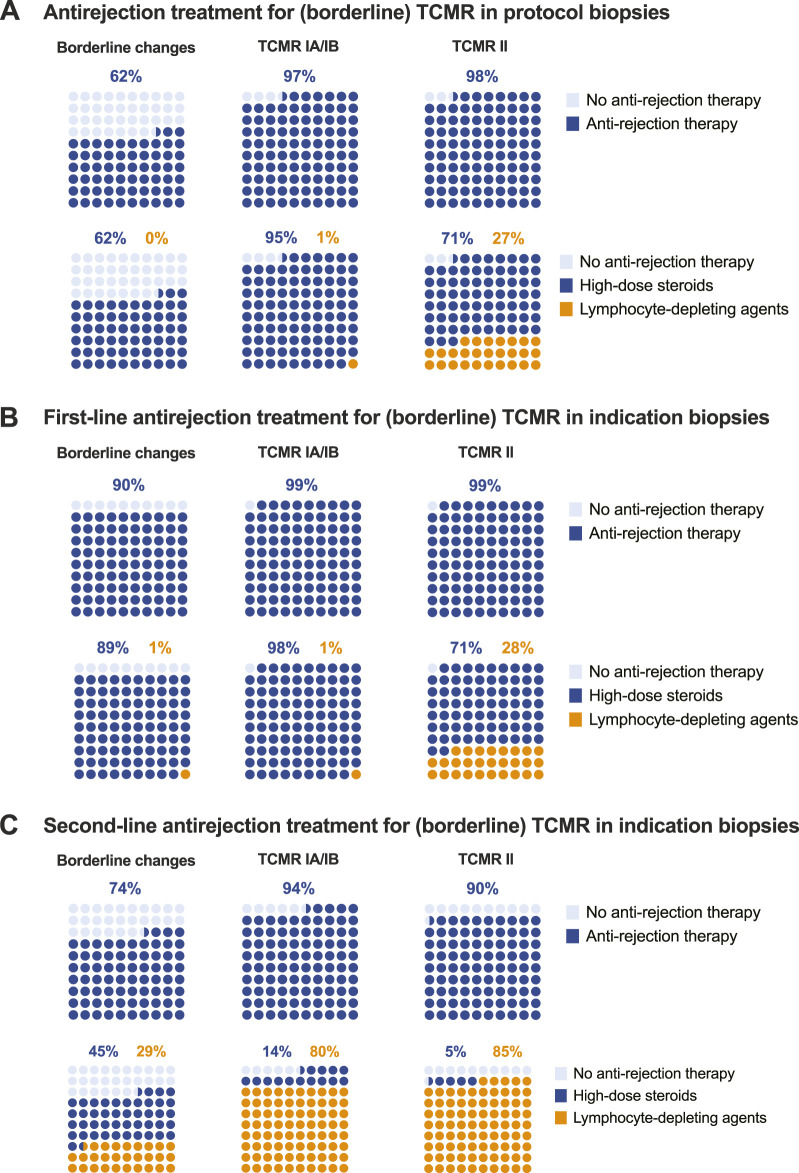
Treatment of TCMR. **(A)** Antirejection treatment for (borderline) TCMR in protocol biopsies. **(B)** First-line antirejection treatment for (borderline) TCMR in indication biopsies. **(C)** Second-line antirejection treatment for (borderline) TCMR in indication biopsies.

Not all centres perform protocol biopsies. Per definition, centres not performing protocol biopsies do not diagnose and do not treat subclinical rejection. Upon detection of subclinical borderline changes, 62.4% of respondents report treating such cases with high-dose steroids, but never with lymphocyte-depleting agents. Other respondents just optimize baseline immunosuppression. Only a small minority reports not changing therapy after the detection of subclinical borderline changes. Most centres treat subclinical TCMR. Treatment of subclinical TCMR consists mainly of high-dose IV steroids, although 27% of respondents report using lymphocyte-depleting agents for treatment of subclinical TCMR grade II (Table 6; Figure 4A).

**TABLE 6:** Treatment of TCMR.

	Protocol biopsies			Indication biopsies		
First-line therapy	Borderline changes	TCMR grade IA/IB	TCMR grade II	Borderline changes	TCMR grade IA/IB	TCMR grade II
Number of respondents	85	85	85	108	108	107
Anti-rejection therapy	53 (62.4%)	82 (96.5%)	83 (97.6%)	97 (89.8%)	107 (99.1%)	106 (99.1%)
Thymoglobulin/ATG/alemtuzumab	0 (0%)	1 (1.2%)	23 (27.1%)	1 (0.9%)	1 (0.9%)	30 (28.0%)
High-dose steroids	53 (62.4%)	81 (95.3%)	60 (70.6%)	96 (88.9%)	106 (98.1%)	76 (71.0%)
- High-dose IV steroids followed by PO taper	7 (8.2%)	23 (27.1%)	24 (28.2%)	16 (14.8%)	28 (25.9%)	32 (29.9%)
- High-dose IV steroids	44 (51.8%	55 (64.7%)	35 (41.2%)	76 (70.4%)	78 (72.2%)	44 (41.1%)
- Steroid taper PO	2 (2.4%)	3 (3.5%)	1 (1.2%)	4 (3.7%)	0 (0%)	0 (0%)
Increased baseline immunosuppression	20 (23.5%)	1 (1.2%)	0 (0%)	11 (10.2%)	1 (0.9%)	1 (0.9%)
No change	12 (14.1%)	2 (2.4%)	2 (2.4%)	0 (0%)	0 (0%)	0 (0%)

Second-line therapy	Borderline changes	TCMR grade IA/IB	TCMR grade II	Borderline changes	TCMR grade IA/IB	TCMR grade II
Number of respondents	—	—	—	98	106	106
Anti-rejection therapy	—	—	—	72 (73.5%)	100 (94.3%)	95 (89.6%)
Thymoglobulin/ATG/alemtuzumab	—	—	—	28 (28.6%)	85 (80.2%)	90 (84.9%)
High-dose steroids	—	—	—	44 (44.9%)	15 (14.2%)	5 (4.7%)
- High-dose IV steroids followed by PO taper	—	—	—	11 (11.2)	6 (5.7%)	1 (0.9%)
- High-dose IV steroids	—	—	—	30 (30.6%)	9 (8.5%)	4 (3.8%)
- Steroid taper PO	—	—	—	3 (3.1%)	0 (0%)	0 (0%)
Increased baseline immunosuppression	—	—	—	17 (17.3%)	4 (3.8%)	8 (7.5%)
No change	—	—	—	9 (9.2%)	2 (1.9%)	3 (2.8%)

#### (Borderline) TCMR in Indication Biopsies

Borderline changes are almost universally treated when diagnosed at the time of graft dysfunction (in indication biopsies). Even more so for TCMR grade I-II, which is universally treated. Lymphocyte-depleting agents are not used as first-line therapy for borderline changes or TCMR grade I, but 28% of respondents report treating TCMR grade II with thymoglobulin, ATG or alemtuzumab in the first line (Table 6; Figure 4B).

Second-line treatment of (borderline) TCMR, after the failure of first-line treatment (with varying definitions), is less universally applied than could be anticipated. This relates especially to borderline changes, where second-line antirejection therapy is not considered in 26.5% of cases, and to TCMR grade II, where 10.4% of respondents would not treat, likely because they already treat these patients with strong therapies (including lymphocyte depleting agents) in first line (Table 6; Figure 4C). Of the 39 respondents proposing lymphocyte-depleting agents as first-line therapy for TCMR grade II, 4 (10.3%) propose alemtuzumab as second-line therapy (after thymoglobulin/ATG); 15 (38.5%) do not propose second-line therapy but just increase baseline immunosuppression after failure of first-line therapy. The other respondents (*N* = 20; 51.2%) repeat the same therapy with lymphocyte-depleting agents despite the lack of success in first-line treatment.

## Discussion

This survey assesses the clinical practices in the transplant centres across Europe for detecting and treating TCMR. A total of 129 participants took part in the survey, wherein the majority were transplant nephrologists with over 11 years of clinical experience, covering the routine clinical practice across all European areas. There were almost equal numbers of small sized transplant centres (centres performing less than 100 kidney transplantations per year) and large sized transplant centres (centres performing 100 to 250 kidney transplantations per year). All conclusions made are against the background of relatively low numbers of centres systematically withdrawing corticosteroids after transplantation, and with a lack of access to, e.g., alemtuzumab in a majority of centres.

The main conclusions of the survey are:

1) Protocol biopsies to detect subclinical rejection are not universally performed, not different between small and larger transplant centres. Some centres always perform protocol biopsies, others never, and still some others only in specific patient populations.
2) The definition of a protocol biopsy is not standardized.
3) The large majority of European centres use classic biomarkers for follow-up after transplantation; donor-derived cell-free DNA assessment or other biomarkers are not used to non-invasively assess the probability of ongoing or future rejection. Sixty percent of centres see BKPyV replication in plasma and *de novo* occurrence of HLA-DSA as indications for a biopsy, but this is also not universal.
4) The most updated Banff Classification is considered as the gold standard for diagnosis of TCMR with also individual Banff lesion scores given, although many respondents are not aware of the detailed thresholds for borderline changes applicable.
5) Biopsy based molecular diagnostics are not commonly used in Europe.
6) There is great heterogeneity in the definition of anti-rejection treatment success. Therapy resistance is sometimes defined based on graft functional evolution, sometimes on histological evaluation of a follow-up biopsy, and often on both together. Systematic control or follow-up biopsies are not common though (and less common than in the US where 40% perform follow-up biopsies [15]); subclinical disease continuation would thus be missed by most European centres.
7) The lack of standardized definition of “baseline graft function” complicates the definition of treatment success, which is often estimated by the total eGFR/creatinine trajectory and not based on a single measurement.
8) There is quite consensus that treatment success or failure is evaluated on a short term, within the first month.
9) Transplant centres consider borderline changes often as indication for therapy, even when diagnosed in protocol biopsies, although not all centres perform such biopsies systematically and subclinical rejection is per definition missed in those centres. Certainly in indication biopsies, borderline changes are deemed clinically meaningful, leading to treatment with high-dose steroids and the related treatment burden/risk.
10) Full TCMR is almost universally treated, with some difference in the approach to TCMR grade I vs. grade II, the latter being treated sometimes with lymphocyte-depleting agents in the first line, although this is the case in only a minority of the centres.
11) Second-line therapy of TCMR consists of a step-up approach towards almost universal use of lymphocyte-depleting agents, if not already used in first line. Centres using lymphocyte-depleting agents in first line (for grade II TCMR) lack efficacious second-line therapies, clear indication of the great unmet need.

Our results about the heterogeneity in the implementation of protocol biopsies are in line with other recent reports [6, 15, 20]. In our survey, respondents indicate that subclinical (borderline) TCMR is treated very similarly to clinical (borderline) TCMR. In Europe, subclinical borderline changes are treated with high-dose steroids in 62% of cases, similar to the 64% reported in Canada [16]. This phenotype is even more often treated in the US with high-dose IV/PO steroids (50%/33%), and even thymoglobulin. Only 22% of subclinical borderline rejections are not being treated in the United States [15], despite lack of evidence of effects on outcome. In case of subclinical TCMR IA and IB, all US centres performing protocol biopsies reported treating this entity, which is comparable to our European survey results and previous Canadian results [15, 16]. Like in Canada, thymoglobulin is virtually not used in Europe for subclinical TCMR grade IA/IB. However, quite some respondents (27%) in Europe propose lymphocyte-depleting agents for subclinical TCMR grade II, again like the practice in the United States [15]. Although performing a biopsy and treating subclinical (borderline) TCMR is not based on strong evidence [10, 12, 21, 22], this indicates that subclinical rejection, when detected and subsequently treated, is a clinically meaningful event, as was also concluded recently by a working group of ESOT [23].

Our survey illustrates that, in Europe, very few centres use innovative non-invasive markers for kidney transplant rejection, and most rely solely on eGFR/creatinine and proteinuria as clinical indication for performing biopsies, while some also see HLA-DSA occurrence and BKPyV replication in plasma as indications for performing a biopsy [24]. At time of graft dysfunction, in indication biopsies, borderline changes is routinely treated in Europe by 90% of respondents using high-dose steroids, even slightly higher than the 81% of the respondents in the US survey who treat this entity using high-dose steroids [15]. This strongly confirms that borderline changes diagnosed at time of graft dysfunction is a clinically meaningful event, potentially suitable as an endpoint for clinical trials [23].

For clinical TCMR IA and IB, all US centres treat with either IV steroids (91%, 71%), PO steroids (21%), or thymoglobulin (13%) [15]. In contrast, thymoglobulin is not often used for this type of rejection in Europe and corticosteroids remain the European mainstay as first-line therapy for this entity, as was also reported for Canada [16]. A final major difference between EU and US is that TCMR grade II is treated with thymoglobulin in 98% of cases in the United States [15], while this is the case for only 28% of respondents in Europe; no data are available for Canada for this rejection type.

Finally, we assessed the definition of successful anti-rejection treatment. The lack of international standardization/consensus on primary definitions hampers the field. Previously, the Canadian survey [16] and an older multicentre survey from 1998 [19], indicated that therapy success is typically measured against the prerejection creatinine level. Our survey adds to this by indicating that most respondents evaluate the overall trajectory of eGFR/creatinine (no single values), and often also integrate information from follow-up biopsies in this evaluation. However, the latter is not at all standardized. Likewise, the Canadian survey indicated that 30% of respondents assessed histological response to treatment independent of changes in kidney function [16]. More systematic study of post-treatment follow-up biopsies would be needed to understand the rate of disease persistence/recurrence despite treatment, which is very likely underestimated according to single-centre data [17, 18].

Notwithstanding the important conclusions of this survey, some limitations are worth mentioning. Not all responses were easily interpretable, especially when “all that apply” multiple choices were allowed (e.g., for definition of steroid/therapy-resistant rejection). We did not assess the baseline immunosuppression or standard induction therapy used by the centres. This study focused on (borderline) TCMR; it remains unclear whether, e.g., repeat biopsies, definition of treatment success/failure, etc. can be generalized also to, e.g., AMR or mixed TCMR-AMR phenotypes. Data analysis remains largely descriptive, and potential relationships between different answers were not systematically assessed.

## Conclusion

In conclusion, our survey indicates that the treatment of TCMR is a great unmet clinical need. Current TCMR treatment is still primarily based on high dose corticosteroids, resembling early transplantation practices. Testing new therapies for TCMR should be in comparison to the current standard of care for TCMR, which differs between the United States and Europe/Canada. Better consensus on treatment success is crucial for robust study designs. However, there is good consensus that treatment success is a short-term outcome parameter, achieved within the first few weeks of/after antirejection treatment. Borderline changes are typically treated like full TCMR, and are thus clinically meaningful when diagnosed in indication biopsies. Subclinical rejections, even borderline changes, diagnosed by some centres performing protocol biopsies, are also often treated despite a lack of robust scientific evidence. The field should investigate innovative treatment options for TCMR after kidney transplantation.

## Data Availability

The original contributions presented in the study are included in the article/Supplementary Material, further inquiries can be directed to the corresponding author.

## Footnotes

1
www.graphpad.com
