# Supplementary material for: European Survey on Clinical Practice of Detecting and Treating T-Cell Mediated Kidney Transplant Rejection
Source: Transpl Int. 2024 Apr 18;37:12283. doi: 10.3389/ti.2024.12283 (PMC11063346; doi:10.3389/ti.2024.12283)
Supplement: Supplementary file 1 [file DataSheet1.pdf]

Supplemental Table 1. Relationship between the number of transplantations per year and performing protocol biopsies. Chi-Square test P = 0.95.

	**Number of transplantations per year**	
**Protocol biopsies**	**<100**	**>100**
Always	35,82%	36,67%
In specific subgroups	22,39%	20,00%
Never	41,79%	43,33%

Supplemental Table 2: Relationship between the % of repeat transplantations and the TCMR rate. Chi-Square test P = 0.049.

	**Percentage repeat transplants**		
**Rate of TCMR**	**<10%**	**11-25%**	**25-50%**
**<11%**	72,73%	39,51%	42,86%
**11-25%**	18,18%	53,09%	42,86%
**>25%**	9,09%	7,41%	14,29%
